# The gut microbiome in anorexia nervosa: relevance for nutritional rehabilitation

**DOI:** 10.1007/s00213-018-5159-2

**Published:** 2019-01-05

**Authors:** Anu Ruusunen, Tetyana Rocks, Felice Jacka, Amy Loughman

**Affiliations:** 1 Deakin University, Food & Mood Centre, IMPACT Strategic Research Centre, School of Medicine, Geelong, VIC Australia; 2 Department of Psychiatry, Kuopio University Hospital, Kuopio, Finland; 3 Institute of Public Health and Clinical Nutrition, University of Eastern Finland, Kuopio, Finland; 4 Murdoch Children’s Research Institute, Melbourne, VIC Australia; 5 Black Dog Institute, Sydney, NSW Australia

**Keywords:** Anorexia nervosa, Eating disorder, Nutritional rehabilitation, Refeeding, Weight restoration, Weight recovery, Malnutrition, Gut microbiome, Gut microbiota

## Abstract

Rapidly accumulating evidence supports the important role of gut microbiome in the regulation of mood, behaviour, appetite, gastrointestinal symptomology, and nutrient metabolism. These are all core features frequently altered in individuals with anorexia nervosa (AN). Current treatment recommendations for AN support the use of high-calorie diets as an essential part of nutritional rehabilitation, commonly achieved by elevating the fat content of the diet. However, in contrast to this approach, there is accumulating evidence suggesting the importance of balanced, high-fibre diets on the gut microbiome. Studies have demonstrated profound differences in the microbial composition of underweight people with AN and those of normal- or overweight individuals. Specific alterations vary widely between studies. It is thus far unclear to what extent the observed differences are brought on by iatrogenic effects of nutritional rehabilitation or the disorder itself. To date, only two studies have investigated the changes in the intestinal microbiota during nutritional rehabilitation and corresponding weight restoration. These studies suggest that the gut microbiome of AN patients was different to healthy controls both prior and following nutritional rehabilitation, though it is noted that these states were associated with lower and higher nutritional intakes, respectively. There is a clear need for further investigation regarding the effects of nutritional rehabilitation on the gut microbiome. Such research would provide insights into the potential role of gut microbiome in modulating the pathophysiology of AN and inform future treatment strategies.

## Introduction

Anorexia nervosa (AN) has poor treatment outcomes and the highest mortality rates of any psychiatric disorder (Arcelus et al. 2011). Currently, closely monitored nutritional rehabilitation and weight restoration is considered the gold standard treatment for AN, surpassing pharmacological interventions and psychotherapy (Bulik et al. 2007; Hay et al. 2014). Previously favoured slow, gradual re-introduction of calories prolongs hospitalisation for AN patients, which has led to the development of higher calorie refeeding protocols (Garber 2017). Faster weight restoration during inpatient treatment (≥ 0.8 kg/week) has been shown to improve the prognosis for later recovery, predicting weight recovery at 1 year (Lund et al. 2009).

The gut microbiome is an under-considered factor in AN pathophysiology and treatment. Since diet is one of the primary influences on gut microbial composition in both the short (David et al. 2014) and long term (Wu et al. 2011), it is likely to be of relevance in AN. Further, the gut microbiome plays an important role in regulating mood (Slyepchenko et al. 2017), behaviour (Dinan et al. 2015), appetite (van de Wouw et al. 2017), gastrointestinal symptomology (Guinane and Cotter 2013), and metabolism (Mithieux 2017). The gut microbiome has been demonstrated to act on the neurobiological, immune, and inflammatory pathways that are implicated in these core and comorbid features of AN via the gut-brain axis (Nguyen et al. 2018; Slyepchenko et al. 2017). It is therefore of great interest to consider the potential role of the gut microbiome of the psychopathology and pathophysiology of AN (Kleiman et al. 2015a).

The primary aim of this review is to summarise the existing literature on the gut microbiome in AN and to consider the potential effects of nutritional rehabilitation on the gut microbiome in this context. A further aim is to inform recommendations regarding the development of future nutritional rehabilitation protocols to better account for their potential impacts on the gut microbiome. The literature search for this narrative review was conducted in June 2018 within PubMed database for articles published in English in the last 20 years using keywords ‘Gut microbiota’, ‘Gut microbiome’, ‘Anorexia nervosa’, ‘Nutritional rehabilitation’, and ‘Weight restoration’. The reference lists of relevant articles were also reviewed with one paper published prior to search limits included.

## The gut microbiome in anorexia nervosa

The human gastrointestinal tract is colonised by an estimated 500–1000 species of bacteria at any one time, as well as archaea, fungi, viruses, and eukaryotes (Turnbaugh et al. 2007). The bacteria are comprised primarily of five phyla: Bacteroidetes, Firmicutes, Actinobacteria, Proteobacteria, and Verrucomicrobia. Firmicutes (including *Lactobacillus*, *Enterococcus*, and *Clostridium* genera) and Bacteroidetes (including *Bacteroides* genus) phyla together represent > 90% of the intestinal community in healthy adults, followed by Actinobacteria and Proteobacteria (Qin et al. 2010). Intestinal microbes participate in digestion and nutrient metabolism, regulate immune function, and have been shown to contribute to disease pathophysiologies (Belkaid and Hand 2014; Hidalgo-Cantabrana et al. 2017; Jandhyala et al. 2015).

Diet composition, particularly the intake of dietary fibre, has been identified as a major determinant of the composition and functions of the microbiome, and correspondingly, of the bacterial metabolites, such as short-chain fatty acids (SCFAs; Ma et al. 2017). Several other factors including host genetics, ethnicity, age, environmental microbial exposures, infections, medications, chronic diseases, stress, physical exercise, and sleep can all affect gut microbial composition (Goodrich et al. 2014; Singh et al. 2017). Thus, there are a number of potential avenues by which the gut microbiome of AN patients may be divergent from that of healthy individuals.

### Microbial diversity and composition in anorexia nervosa

Microbial diversity and composition in AN patients has been demonstrated to differ from healthy individuals and overweight/obese controls in almost all reviewed studies (Table 1). Total count of bacteria and, for example, of butyrate-producers, such as *Roseburia*, have been found to be reduced compared to controls. In contrast, the abundance of mucin-degrading bacteria such as *Akkermansia muciniphila* and archaeon *Methanobrevibacter smithii* has been shown to be increased. However, the level of detail regarding gut microbiota results differs widely across the studies and reported microbiome compositional differences are not consistent.

**Table 1:** Studies demonstrating the gut microbiota in anorexia nervosa (AN) patients and reporting potential differences between patients and controls

Author(s)	Country	Study populations	Body mass index (kg/m^2^) of the participants ± SD (range)	Other characteristics of AN participants	Faecal sample collected in patients (prior, during, or post-treatment)	Reported differences in MB composition: AN patients compared to controls	Other results
Armougom et al. (2009)	France	AN patients (*n* = 9) (DSM-IV criteria)	12.7 ± 1.6	Age: range 19–36 years Sex: female	AN patients recently hospitalised; no other details of sample collection provided	↑ Archeon *Methanobrevibacter smithii* No differences: Firmicutes, Bacteroidetes, and *Lactobacillus* similar to lean controls	In obese individuals, bacterial profiles were poor in Bacteroidetes and rich in *Lactobacillus*
		Lean/normal-weight controls (*n* = 20)	20.7 ±2.0				
		Obese controls (*n* = 20)	47.1 ± 10.7				
Pfleiderer \et al. (2013)	France	Case study (*n* = 1) No controls	10.4	Age: 21 years Sex: female History: suffered from a severe restrictive AN for 9 years. No remission, BMI had been fluctuating between 10 and 15 kg/m^2^. Diet: vegetables, fruits, dairy	A single faecal sample at the day of hospitalisation before nutritional rehabilitation started	–	19 bacterial species never previously isolated from human gut were found, including 11 new bacterial species in the phyla Firmicutes, Bacteroides, and Actinobacteria
Million et al. (2013)	France	AN patients (*n* = 15) (DSM-IV criteria) Normal-weight controls (*n* = 76)	13.5 (11.7–14.6) 22.4 (20.7–23.7)	Age: 27.3 ± 10.8 years Sex: 14 females, 1 male	Faecal samples collected from hospitalised and outpatients (timing N/A)	↓ *Lactobacillus reuteri* compared to all controls ↑ *Escherichia coli* compared to obese controls ↑ Archeon *Methanobrevibacter smithii* compared to obese controls	Faecal levels of *Lactobacillus reuteri* positively correlated and *E*. *coli* negatively correlated with BMI in all study groups
		Overweight controls (*n* = 38)	27.1 (25.9–28.6)				
		Obese controls (*n* = 134)	40.0 (36.4–46.8)				
Gouba et al. (2014) (Reporting on same patient as Pfleiderer et al. (2013))	France	Case study (*n* = 1) No controls	10.4	Age: 21 years Sex: female History: a severe AN with severe malnutrition Diet: vegetables, fruits, fruit juices, dairy, cereals, rice, nuts, legumes, fish, turkey	A single faecal sample at the day of hospitalisation before nutritional rehabilitation started	–	4 new microeukaryote species not previously detected in human faecal samples, and low diversity of fungi identified
Kleiman et al. (2015b)	USA	AN patients (*n* = 16) (DSM-IV-TR criteria)	16.2 ± 1.5 (admission)	Age: 28.0 ± 11.7 years Sex: female	A single faecal sample produced after admission (at the visit or at home and brought to the visit) and one prior to discharge	↓ Microbial alpha-diversity ↑ Bacilli ↑ Coriobacteriales↓ Phylogenetic richness ↓ Clostridia ↓ *Faecalibacterium* ↓ *Anaerostipes*	Depressive symptoms were inversely associated with the number of observed bacterial species and Chao-1 estimator. Higher eating disorder psychopathology was associated with lower number of observed species. After treatment: Alpha-diversity and microbial richness increased. Changes at the phylum and genus levels were demonstrated compared to admission, for example ↑ *Ruminococcus* spp. Alpha-diversity remained lower in post-treatment AN compared to controls
		Healthy controls (*n* = 12) (no recurring GI symptoms)	17.4 ± 0.9 (discharge) 21.5 ± 1.9				
Morita et al. (2015)	Japan	AN patients (*n* = 25) (AN-R *n* = 14, and AN-BP, *n* = 11)	12.8 ± 1.3	Age: 30.0 ± 10.2 years Sex: female	Patients admitted as inpatients or visited outpatients. Faecal samples collected into two tubes by the participants or hospital staff, timing not reported	↓Total count of bacteria and obligate anaerobes (*Clostridium coccoides* group, *Clostridium leptum* subgroup, *Bacteroides fragilis*, *Streptococcus*) *↓* Abundance of *Lactobacillus plantarum* ↓ SCFAs (acetatic acid and propionic acid) in faeces	No significant differences between AN-R and AN-BP in genus or family level. *Clostridium difficile* was only detected in the AN-BP group, not in the AN-R or control group
		Age- and sex-matched normal-weight controls (*n* = 21)	20.5 ± 2.1				
Mack et al. (2016)	Germany	AN patients (*n* = 55) Normal-weight controls (*n* = 55)	15.3 ± 1.4 (11.6–17.7) At discharge: 17.7 ± 1.4 (13.9–21.8). 21.6 ± 2.0 (18–25)	Age: 23.8 ± 6.8 Sex: female	Faecal samples collected as soon as possible after the beginning of inpatient care and one prior to discharge	↓ Number of OTUs and the Chao1 index in AN patients who used laxatives ↑Mucin-degrading bacteria (including *Verrucomicrobia*, *Bifidobacteria*) ↑ Branched-chain fatty acids and valerate ↓Butyrate-producers (e.g., *Roseburia*) No differences: SCFAs	After weight gain, microbial richness and diversity (the Shannon index) increased in patients (*n* = 44) relative to controls. Lower GI symptoms, but not upper GI symptoms, improved during treatment. Unspecified differences in microbial community structure between AN-R and AN-BP
Borgo et al. (2017)	Italy	AN patients (*n* = 15)	13.9 ± 2.1	Age: not reported Sex: females	Timing of the sample collection not reported	↑ Gram-negative bacteria ↑ Proteobacteria ↑ *Enterobacteriaceae* ↑ Archeon *Methanobrevibacter smithii* ↓ Firmicutes ↓ Ruminobacteria ↓ *Roseburia* ↓ *Ruminococcus* ↓ *Clostridium* *↓* Total SCFAs, propionate, butyrate No differences**:** Phylogenetic diversity, phylogenetic richness, beta-diversity, faecal acetate, isovalerate and isobutyrate	Faecal butyrate concentrations inversely correlated with depression and anxiety symptom severity. BMI had a negative correlation with *Bacteroides uniformis* and psychopathological scores (obsession-compulsion, anxiety, depression)
		Age- and sex-matched controls (*n* = 15)	22.1 ± 2.6				
Kleiman et al. (2017)	USA	AN patients (*n* = 3) No controls	15.6, 17.6, and 13.7 at admission; 20.2, 21.1, and 15.4, respectively, at discharge. Durations of the data collection were 73, 58, and 34 days, respectively	Age: 25 years, 29 years, and 16 years Sex: females	Faecal samples were collected on a daily basis (or as frequently as possible) by unit nurses/assistants trained in collection protocols or after getting to the partial hospitalisation program, at home	–	Significant patient-specific changes in composition and diversity over time observed at the phylum (*n* = 4), class (*n* = 8), order (*n* = 14), family (*n* = 28), and genus (*n* = 68) levels. REE increased during treatment, in parallel with energy intake and BMI. REE was not significantly related to composition or diversity of gut microbiota. Diet-induced thermogenesis reached a peak after 2–3 weeks of treatment
Mörkl et al. (2017)	Austria	AN patients (*n* = 18)	15.3 ± 1.3	Age: 22.4 ± 3.2 Sex: females	Stool samples were collected at the beginning of an inpatient hospital stay	↑ Coriobacteriaceae ↓ Alpha-diversity ↓ Microbial richness only compared to athletes	Alpha-diversity was negatively correlated with depression scores (BDI) when all groups were included in the analysis, but not in AN patients only
		Athletes (*n* = 20)	22.1 ± 1.8				
		Normal-weight controls (*n* = 26)	21.9 ± 1.7				
		Overweight controls (*n* = 22)	27.0 ± 1.1				
		Obese controls (*n* = 20)	34.6 ± 4.4				

*AN*, anorexia nervosa; *AN*-*R*, anorexia nervosa restrictive; *AN*-*BP*, anorexia nervosa binging-purging; *BDI*, Beck Depression Inventory; *BMI*, body mass index; *DSM*, Diagnostic and Statistical Manual of Mental Disorders; *GI*, gastrointestinal; *N*/*A*, not available; *REE*, resting energy expenditure; *SCFA*, short-chain fatty acid; *SD*, standard deviation

In general, a reduction in microbial diversity is associated with impaired immune defence and reduced capacity for harvesting calories from the diet (Lippert et al. 2017). Although some differences have been observed in microbial diversity and richness (alpha-diversity) in AN patients, the number of observed species and Chao 1 index, a measure of alpha-diversity, in patients were comparable to those of normal-weight controls in the largest study in this field conducted in Germany (*n* = 55 AN patients; Mack et al. 2016). In this study, most of the patients were reported to consume fruit, vegetables, and whole-wheat bread on a daily basis prior to treatment (Mack et al. 2016). This was reflected in daily fibre intake comparable to normal-weight controls at the time of hospital admission. The percentages of calorie intake derived from macronutrients were also within the standard dietary guidelines, despite the AN patients’ total calorie intake being low. The aforementioned characteristics of the diet, especially relatively high fibre intake, in this study may have protected against the expected reduction in the alpha-diversity of gut microbiota in this sample (Mack et al. 2016).

Bacteroidetes, Firmicutes and, to a lesser extent, Actinobacteria, Proteobacteria, and Verrucomicrobia have been found to be the dominant phyla found in the gut microbiome of AN patients, similar to those in normal-weight controls (Borgo et al. 2017; Mack et al. 2016). Generally, weight loss by low-carbohydrate or low-fat diets seems to lead to increased levels of Bacteroidetes (Fava et al. 2013), whereas high-fat diets are associated with increased levels of both Firmicutes and Proteobacteria and a reduction of Bacteroidetes (Murphy et al. 2015). A reduction of calorie intake and weight either with a fat-restricted or a carbohydrate-restricted low-calorie diet has been shown to increase the relative abundance of Bacteroidetes, which suggests that calorie restriction may be more relevant to Bacteroidetes levels than calorie source (Ley et al. 2006). However, the results of studies examining the relative abundance of Firmicutes and Bacteroidetes in AN patients have been contradictory. Whilst the Firmicutes-to-Bacteroidetes ratio was significantly increased in one study (Mack et al. 2016), showing a similar trend to what is described in obese individuals, the direction of this finding was inverted in AN patients in another study (Borgo et al. 2017). Previous animal studies have also suggested a key role of gut microbiota in calorie harvest and extraction of calories from food (Backhed et al. 2004), which indicates a direct role of gut bacteria in nutrient absorption, potentially relevant in AN. In lean human individuals, excess intake of calories (up to 3400 kcal/day) prompted a change in the Firmicutes-to-Bacteroidetes ratio (Jumpertz et al. 2011). This in turn increased the degree to which calories were harvested from nutritional intake by up to 150 kcal/day (Jumpertz et al. 2011).

Patients with AN have been found to have elevated relative abundance of Actinobacteria (mainly *Bifidobacteria*) (Mack et al. 2016) and elevated levels of *Proteobacteria* and *Enterobacteriaceae* (Borgo et al. 2017) compared to normal-weight controls. AN patients have also demonstrated lower abundance of *Lactobacillus* (Armougom et al. 2009; Million et al. 2013) and decreased levels of the carbohydrate-fermenter *Ruminococcus* (Borgo et al. 2017) and butyrate-producing *Roseburia* (Borgo et al. 2017; Mack et al. 2016). *Roseburia* levels seem to correlate with butyrate levels in AN patients (Mack et al. 2016). AN patients have also demonstrated increased levels of faecal *Coriobacteriaceae* (Mörkl et al. 2017). Interestingly, higher levels of *Coriobacteriaceae* have similarly been identified in humans after endurance exercise (Zhao et al. 2018). Moreover, two exploratory case studies in people with AN (Gouba et al. 2014; Pfleiderer et al. 2013) showed 11 completely new bacterial species and four new micro-eukaryote species, respectively, in a single faecal sample, which points to the possibility of atypical conditions in the intestinal tract of individuals with AN.

Several studies in AN patients have shown elevated levels of archaea, with the main genus of interest being *Methanobrevibacter smithii*, a methane-producing archaeon (Armougom et al. 2009; Borgo et al. 2017; Mack et al. 2016; Million et al. 2013). In a German study (Mack et al. 2016), 22% of AN patients (compared to 15% of normal-weight controls) were found to carry *Methanobrevibacter smithii*, whereas it was observed in 100% of AN participants in the French study (Armougom et al. 2009), which was more common than in lean (75%) or obese (80%) participants. *Methanobrevibacter smithii* plays an important role in improving the efficiency of microbial fermentation, and its abundance has been hypothesised to optimise calorie extraction from a diet with very low calorie content, allowing extra calories to be extracted (Armougom et al. 2009; Carr et al. 2016). Elevated prevalence of methane-producing bacteria has also been shown in individuals who suffer from constipation (Fiedorek et al. 1990), which is a known complication of AN.

Finally, two studies have compared intestinal microbial compositions between restrictive (AN-R) and binging-purging subtypes of AN (AN-BP) (Mack et al. 2016; Morita et al. 2015). AN-R and AN-BP subtypes differ significantly in their eating behaviour, as individuals with AN-BP occasionally eat large amounts of foods at one time, which is often followed by vomiting. The two studies report divergent findings: there were no significant differences between AN-R and AN-BP in terms of abundances of individual species in the Japanese study (Morita et al. 2015), whilst the microbial community structure was significantly explained by AN subtype in the German study (Mack et al. 2016). It is noted, that there are many statistical options for analysing the microbiome, and the choice of test may explain some of the divergence found in these and other microbiome studies.

### Microbial metabolites

Unlike other carbohydrates, non-digestible carbohydrates are not enzymatically broken down in the small intestine but are fermented by intestinal microbes in the large intestine (Singh et al. 2017). This microbial fermentation of non-digestible carbohydrates, fibre and resistant starch, results in the formation of SCFAs, which fuel epithelial cells in the colon and influence immune responses and epithelial integrity (David et al. 2014; Singh et al. 2017). Levels of total SCFAs, acetate, propionate, and butyrate, are inconsistently reported in AN studies. SCFA levels were found to be comparable between AN patients and normal-weight participants in the German study (Mack et al. 2016), but reduced in other studies (Borgo et al. 2017; Morita et al. 2015). In the Japanese study (Morita et al. 2015), acetate and propionate concentrations were decreased in this group, whilst in the Italian study, both total SCFAs, butyrate and propionate levels, were reduced (Borgo et al. 2017). In contrast, in the German study (Mack et al. 2016), only butyrate proportions were slightly lowered in AN patients compared to normal-weight controls. As the evidence is inconsistent to date, the role of SCFAs in the onset and progression of AN needs further investigation.

Branched-chain fatty acids (BCFAs) are products of protein fermentation by colonic microbiota and are mainly formed from the branched-chain amino acids (BCAAs), such as valine, isoleucine, and leucine (Macfarlane et al. 1992). Concentrations of total BCFAs, in particular valerate and isobutyrate, are increased in AN patients at hospital admission (Holman et al. 1995; Mack et al. 2016), indicating increased protein fermentation in the gut. Animal-based diets may promote BCAA metabolism by colonic bacteria (David et al. 2014), and the BCFAs isobutyrate and isovalerate are particularly pronounced on a high-protein, animal-based diet (Russell et al. 2011). It is also suggested that other protein fermentation metabolites (such as phenols, ammonia, and amines) could contribute to the detrimental impact of malnutrition on gut physiology and motility (Mack et al. 2016). However, the role of SCFA and BCFA levels in relation to both non-fermentable carbohydrate and fat and protein intake in AN needs further investigation.

### Associations of altered microbial composition with weight and starvation in anorexia nervosa

Microbial composition plays a general role in weight regulation in both humans and animals, although the extent of its contribution is still controversial (Carr et al. 2016). In AN, *Bacteroides uniformis* (Borgo et al. 2017) and *Escherichia coli* (Million et al. 2013) were negatively associated with body mass index (BMI), whilst *Lactobacillus reuteri* was positively associated with BMI (Million et al. 2013). *L*. *reuteri* was relatively rare in both AN (BMI < 14.6) and lean (BMI 14.6–23.7) individuals, detected in only 7% and 8% of individuals, respectively, compared to 22% in obese individuals (Million et al. 2013). In contrast, in a case-series study with three AN patients, no evidence of strong associations between the composition of intestinal microbiota and BMI was observed despite significant weight gain during the treatment (Kleiman et al. 2017). Inter-individual variability is a significant limitation to the generalisability of such small sample studies. BMI is positively associated with SCFA concentrations (total SCFAs, butyrate, propionate, and isobutyrate) in AN patients (Borgo et al. 2017), a finding which has also been reported in obese individuals (Fernandes et al. 2014).

Elevated levels of mucin-degrader Verrucomicrobia (mainly *Akkermansia muciniphila*) have been demonstrated in AN patients relative to healthy controls (after the exclusion of laxative users from the analyses) (Mack et al. 2016). *Akkermansia muciniphila* levels are positively associated with weight-loss and inversely associated with body weight (Isokpehi et al. 2017). *Akkermansia muciniphila* is a bacterium living within the gut mucus layer, which obtains nutrients from the layer (Mithieux 2017), and is identified as a key mucin degrader (Derrien et al. 2004). Its abundance has been associated with being in a state of fasting (Marcobal et al. 2013), but also can be increased with elevated fibre intake (Everard et al. 2013). As higher levels of Verrucomicrobia (especially *Akkermansia* spp.) have also been found in fasting and hibernation studies in animals, higher levels may be related to fasting, not to the AN phenotype itself (Mack et al. 2018).

A recent review on the impacts of starvation on gut microbiota across both human and animal studies (Mack et al. 2018) concluded that the directionality of this relationship remains complex and unclear. There are indeed a number of plausible biological mechanisms by which the gut microbiome could affect appetite, satiety, and eating behaviour, all factors affecting starvation. For example, the production of SCFAs via the microbiome may facilitate the secretion of satiety hormones (peptide YY and a glucagon-like peptide) expressed by gut enteroendocrine cells (Alcock et al. 2014). Also connected to satiety, *Enterobacteriaceae*, particularly the *Escherichia coli* species, could have a role in AN through neuropeptide caseinolytic protease b (ClpB) (Breton et al. 2016). *Enterobacteriaceae* are found to produce ClpB, which in turn is an anorexigenic protein also known to have anxiolytic properties. ClpB production correlates with α-melanocyte-stimulating hormone levels, which are known to be involved in satiety and anxiety signalling in eating disorders (Adan and Vink 2001). Borgo and colleagues (Borgo et al. 2017) hypothesised that higher abundance of gram-negative bacteria, especially *Enterobacteriaceae*, could be connected to elevated production of ClpB, which in turn could mediate gut-brain communication in AN.

### Mood, anxiety, and eating disorder psychopathology

There is evidence supporting a role for the gut microbiome in the regulation of mood and anxiety (Slyepchenko et al. 2017). Comorbidity between depression, anxiety, and AN is significant; about two thirds of AN patients experience onset of major depressive disorder in the same year or in the year following diagnosis of AN, and up to 81% of AN patients experience major depressive disorder at some point in life (Fernandez-Aranda et al. 2007). Similarly, up to 72% of AN patients experience one or more anxiety disorders during their lifetime (Godart et al. 2002).

Only two studies have investigated the association between gut microbial composition and psychopathology in AN patients (Borgo et al. 2017; Kleiman et al. 2015b. In these studies, elevated depressive and anxiety symptoms were significantly more common in AN patients than in healthy controls (Borgo et al. 2017), 80% and 67% for each respective symptom type in AN (Kleiman et al. 2015b). BMI was inversely associated with eating disorder psychopathology, including obsessive-compulsive symptoms, depression, and anxiety. The number of observed bacterial species and stool butyrate levels were inversely associated with depressive symptoms (Borgo et al. 2017; Kleiman et al. 2015b). SCFAs, such as butyrate, are suggested to enhance colonic production and secretion of serotonin (Reigstad et al. 2015). Moreover, a negative correlation was observed between depression scores and *Clostridium* spp. (Borgo et al. 2017). Several antidepressant medications, such as sertraline, fluoxetine, and paroxetine, also have antimicrobial effects (Munoz-Bellido et al. 2000); however, these medications were not accounted for in the described studies. Due to limited reports of comorbidities and medications, it is unclear whether any of the described changes in gut microbiota were influenced by these factors. In addition, it is worth considering that evidence of the role of gut microbiome in the psychopathology of AN thus far consists of correlation studies, and no mechanistic studies are yet available.

### Compensatory behaviours

Laxatives are used both in the treatment of short-term and prolonged constipation in AN, but one third of AN patients also use laxatives as a compensatory behaviour (Elran-Barak et al. 2017). The overall number of species and estimated richness was shown to be particularly reduced in those AN patients who had a history of laxative use (Mack et al. 2016). Studies describing high-dose, acute laxative use, such as via pre-surgical bowel preparations, have shown detrimental alterations in intestinal microbiota (Jalanka et al. 2015). Although the microbial community restores itself over time, the continuous use of laxatives may threaten the long-term balance of commensal bacteria. In healthy adults, previous research demonstrated that such a restoration occurred within 2 weeks. However, the rate of this recovery is dependent on the dose of laxatives and initial composition of microbiota (Jalanka et al. 2015). Furthermore, effects of long-term, regular use of laxatives, as commonly reported in AN populations, on the gut microbiome have yet not been established.

## Nutritional rehabilitation procedures and gut microbiome

Nutritional rehabilitation protocols based on high caloric intake prioritise rapid weight gain, and these have been shown to be safe and efficient for achieving weight restoration (Peebles et al. 2017; Smith et al. 2016). High-calorie refeeding protocols are now the standard of care in AN, with 85% of recent studies (published between years 2010 and 2015) commencing nutritional rehabilitation with at least 1400 kcal/day and proceeding with rapid increments up to high-calorie diet (Garber et al. 2016). Effective early weight gain is considered a positive predictor of recovery and future remission, which supports the change to protocols that promote faster weight gain in malnourished AN patients (Le Grange et al. 2014; Madden et al. 2015; Peebles et al. 2017). However, long-term effects on recovery and overall health of rapid changes in adiposity, particularly central adiposity, are yet to be confirmed (El Ghoch et al. 2015). Furthermore, none of the published high-calorie refeeding protocols have been tested for their possible effects on the gut microbiome.

Nutritional rehabilitation diets for weight restoration in AN are commonly high in fat (Mack et al. 2016), as fat is the most calorie-dense nutrient; however, the exact macronutrient distributions are rarely described. Consuming a greater proportion of total calories from fat leads to improved weight recovery in AN patients (Baskaran et al. 2017). However, proportions of macronutrients, such as fats, proteins, and carbohydrates (including fibre), as well as overall calorie intake, can significantly alter the composition of the gut microbiota (Scott et al. 2013). In particular, diets high in fat and protein and low in non-digestible carbohydrate and other fibre may lead to altered microbial diversity and potential dysbiosis (De Filippo et al. 2010; Simpson and Campbell 2015; Singh et al. 2017). In animal models, high-fat diets are consistently shown to increase gut permeability and circulate inflammation (Cani et al. 2008). Importantly, different types of dietary fats appear to have differential effects on the microbiota, suggesting that whether monounsaturated, polyunsaturated, or saturated fats are included in the dietary protocols, as well as the foods from which they are derived, could be of substantial importance to gut microbiota outcomes (Fava et al. 2013; Huang et al. 2013). Saturated fats are seen to particularly induce gut permeability and insulin resistance (Lam et al. 2015), as well as neuroinflammation (Valdearcos et al. 2014), whereas monounsaturated fats, particularly when combined with phenolic compounds (Martin-Pelaez et al. 2017), and polyunsaturated fats from fish (Caesar et al. 2015) appear to be protective. Therefore, the specific composition of rehabilitation diet may influence the composition of the gut microbiome.

### Effects of nutritional rehabilitation on gut microbiome

Only two studies in AN inpatients have reported changes in gut microbiome during nutritional rehabilitation and weight restoration (Kleiman et al. 2015b; Mack et al. 2016). In the first, an American study with 10 AN inpatients (Kleiman et al. 2015b), microbial richness, characterised by the number of observed bacterial species and the Chao-1 estimator of diversity, increased during nutritional rehabilitation and weight gain. However, when diversity of the post-rehabilitation intestinal microbiome was compared to normal-weight controls, decreased alpha-diversity was still apparent in those recovering from AN (Kleiman et al. 2015b). The study reported shifts to microbial composition following nutritional rehabilitation as measured by unweighted UniFrac distances and trends toward changes to the relative abundance of the bacteria from the *Coccaceae* family (Kleiman et al. 2015b). Whilst these findings provide some evidence of the impact of nutritional rehabilitation on the gut microbiome, it is difficult to draw strong conclusions since the details of the nutritional rehabilitation procedures were not provided.

In the second relevant study, microbial diversity and richness in AN patients at baseline was comparable to that of controls (Mack et al. 2016). Diversity, as measured by the Shannon index, significantly increased during the course of weight gain. Despite this post-treatment increase in diversity, the AN group’s microbiota remained more similar to its own baseline microbiota than to that of the healthy control group (Kleiman et al. 2015b; Mack et al. 2016).

Mack et al. (2016) also reported a post-rehabilitation increase of Firmicutes and decrease of Bacteroidetes phyla, which could be a result of the high-caloric diet, as high-fat diets have previously been associated with changes in the ratio of Firmicutes-to-Bacteroidetes (Murphy et al. 2015). No changes to these phyla were reported in the American study (Kleiman et al. 2015b). However, *Ruminococcus* levels increased over the course of weight gain in both of these studies (Kleiman et al. 2015b; Mack et al. 2016). This increase may reflect the increased intake of fibre and resistant starch (Ze et al. 2012), as also suggested by the German study (Mack et al. 2016). In their study, fibre intake was relatively high (25–33 g/day) during nutritional rehabilitation (Mack et al. 2016). In addition, reduced levels of carbohydrate-utilising taxa (especially *Roseburia*, which produces butyrate) and elevated relative abundances of mucin and protein-degrading taxa were found at genus level in AN patients after nutritional rehabilitation (Mack et al. 2016). Finally, the abundance of *Methanobrevibacter smithii* decreased from 22% of AN patients at hospital admission to 14% after nutritional rehabilitation, which was comparable to healthy controls (15%) (Mack et al. 2016).

Mack and colleagues also reported that alterations in faecal SCFA concentrations (especially low butyrate proportions) did not recover in AN patients following treatment (Mack et al. 2016). SCFAs have been postulated to provide an additional ≤ 10% of total daily calorie intake in humans (Bergman 1990). Moreover, after nutritional rehabilitation, high BCFA concentrations, especially total BCFA and valerate concentrations, were found to further increase after weight restoration (Mack et al. 2016), which indicates increased protein fermentation. Interestingly, a shift away from SCFA production and toward amino acid fermentation has also been demonstrated following weight-loss surgery, thought to be due to the resulting reduced calorie harvest from dietary intake (Tremaroli et al. 2015).

### Feeding procedures

Weight restoration in AN is conducted via foods administered within a standard or individualised meal plan, and also using special nutrition supplements and/or nutrition delivered via nasogastric feeding to ensure the adequate intake of nutrients (Kells and Kelly-Weeder 2016). Exclusive enteral nutrition has been shown to cause alterations in gut microbiota, as well as leading to reduced SCFA production (Berntson et al. 2016), which may at least in part be explained by the low fibre content in enteral formulas. Enteral formulas consist of ingredients, such as triglycerides and corn syrup, and various synthetic substances, including dietary emulsifiers (Krezalek et al. 2017). Dietary emulsifiers have been suggested to promote detrimental changes to the gut microbiome, gut permeability, and intestinal inflammation and are associated with elevated levels of anxiety (Holder and Chassaing 2018). Probiotics, on the other hand, have been suggested to be beneficial in improving immune function and inflammatory response during nasogastric feeding (Xie et al. 2018).

In rare cases, parenteral nutrition may provide life-saving support for AN patients when adequate calorie intake is not possible through eating. It is postulated that parenteral nutrition also disrupts the normal microbiome and may explain the observed impaired immune function and epithelial barrier observed in long-term intravenous feeding (Pierre 2017). However, none of the studies describing gut microbiota in AN patients have reported the presence of nasogastric or parenteral feeding in their study populations (Table 1). Also, the studies describing the effects of total enteral or parental feeding have not included AN patients (Krezalek et al. 2017; Pierre 2017). Therefore, the potentially important effects of these nutritional rehabilitation methods on gut microbiota remain unstudied.

### Gastrointestinal symptoms

Gastrointestinal symptoms are highly prevalent amongst AN patients and may also be affected by changes in eating and the gut microbiome during the course of treatment. Nutritional rehabilitation has been shown to decrease lower gastrointestinal symptoms (e.g., constipation) but not upper gastrointestinal symptoms (e.g., abdominal fullness, abdominal bloating, and feeling of abdominal distension) in this population (Mack et al. 2016). Furthermore, abdominal pain and feeling of incomplete evacuation have still been more common in AN patients compared to controls. A diet high in fibre as well as high in fat could potentially lead to improved gastrointestinal symptoms since the increase of dietary substrates would create advantageous conditions for higher abundance of carbohydrate utilising and mucin-degrading taxa (Mack et al. 2016). However, there are currently no dietary interventions focusing on improving GI symptoms in AN patients, targeting the gut microbiome or otherwise.

### Physical activity

AN patients are usually obliged to limit physical activity in order to reduce total energy expenditure rates and promote weight recovery during nutritional rehabilitation. Physical exercise is, however, known to benefit gut health and microbial composition (Monda et al. 2017). For example, physical activity has been associated with increased alpha-diversity (Mörkl et al. 2017) and in anorexic mice, physical activity during refeeding improved colonic permeability and was also positively associated with body composition (Achamrah et al. 2016a). Additionally, although it still remains unclear if adapted physical activity during nutritional rehabilitation is beneficial for the restoration of body composition in AN patients, physical activity is known to be beneficial for depression, anxiety, and preservation of bone mineral density; these are all frequent comorbidities of AN (Achamrah et al. 2016b). Supervised physical exercise appeared to be safe and to have no detrimental effects on weight recovery and to have beneficial effects on cardiovascular fitness (Ng et al. 2013). Thus, AN patients may benefit from physical activity as a personalised therapeutic approach that may reinforce physical health (Achamrah et al. 2016b), although potential beneficial effects on gut microbiota are yet to be confirmed.

## Considerations for treatment

Current evidence suggests that a diet favourable for gut health should be high in non-digestible carbohydrates, diverse in fibre subtypes, with adequate amounts of good quality protein (primarily plant-based) and healthy fats (mono- and polyunsaturated fatty acids) (David et al. 2014; Scott et al. 2013; Singh et al. 2017). High-calorie diets are required in AN; however, it may be beneficial to combine high-fat with high-fibre in order to promote a gut microbial composition akin to that of healthy samples. Similarly, it is likely important to ensure that the fat component of the diet comes from mono- or polyunsaturated fats that have demonstrated benefits to the gut microbiome and metabolic health, rather than from saturated fats. However, it is noteworthy that exposure to large variety of foods is an essential part of AN treatment. These may also include foods considered ‘unhealthy’ from gut microbiome’s perspective, such as foods high in saturated fats or sugar, but considered ‘healthy’ from psychological perspective, acting as an important exposure to address fear of eating. Therefore, some of these foods should remain a part of nutritional rehabilitation.

Promoting high-fibre diet during nutritional rehabilitation in AN may be challenging in practice, as additional fibre intake means increased volumes of low-energy foods and elevated satiety. Therefore, it is of particular importance to aim to include small amounts of beneficial fibres in every meal or snack provided. This gradual approach will also be important in managing bowel symptoms that may arise from the introduction of high-fibre foods in AN patients. In practice, this may translate to consumption of small amounts initially, with continuous incremental increase in high-fibre foods to supplement high-fat, high-protein foods in order to support high calorie intake. However, the benefits of high-fibre refeeding protocols should be considered against the immediate needs of AN patients, and careful individualised considerations should be paramount. However, as the nutritional rehabilitation progresses, additional fibre intake should be a focus to facilitate more favourable recovery of the gut microbiome. With increasing evidence in gut microbiota research in general health and disease—and in AN in particular—personalised refeeding protocols may eventually be possible and necessary, given the demonstrated individual variations in the human gut microbiome.

To date, there is no AN-specific evidence to recommend prebiotic or probiotic supplementations. However, rapidly emerging evidence suggests that non-digestible carbohydrates and prebiotic foods have an important role in generating SCFAs, as well as elevating the levels of beneficial intestinal *Bifidobacteria* and lactic acid bacteria (Cotillard et al. 2013; Simpson and Campbell 2015; Singh et al. 2017). Furthermore, based on an animal research, supplementation with *Bifidobacteria* leads to elevations in the serotoninergic precursor tryptophan, suggesting a possible role for probiotics in modulation of neurotransmitter levels (Desbonnet et al. 2008). Dietary sources of prebiotics include non-digestible carbohydrates, in the form of rye, wheat, barley, oat, and legumes, and non-digestible oligosaccharides, such as inulin, fructans, polydextrose, fructo-oligosaccharides, and galacto-oligosaccharides (Pandey et al. 2015). Higher intake of non-digestible oligosaccharides, including inulin and oligo-fructose, and resistant starch is associated with positive health outcomes (Scott et al. 2013; Singh et al. 2017) and can be utilised to modify the intestinal environment (Singh et al. 2017), promote growth of beneficial species, reduce pH, and also help pathogen exclusion (Scott et al. 2013). Resistant starch, from sources such as green bananas, lentils, beans, and cooked-then-cooled potatoes and rice, has also been found to increase abundance of beneficial carbohydrate-fermenters *Ruminococcus* and *Roseburia* (Singh et al. 2017; Walker et al. 2011). However, as noted previously, intakes of some foods that are good sources of prebiotics and resistant starch may induce unfavourable gastrointestinal symptoms. Therefore, these foods should be introduced slowly in AN patients and tailored to individual tolerance.

Future nutritional rehabilitation protocols in AN are encouraged to include higher and more diverse fibre content and prebiotic foods. This may provide benefits on intestinal microbes and their metabolites and prevent gastrointestinal sequelae, especially constipation (Mack et al. 2016). Similarly, studies to investigate the potential role of probiotic foods or supplementations as a part of nutritional rehabilitation procedures are needed in the future. For example, butyrate-producing bacteria, namely *Roseburia* spp, have been suggested as candidates for probiotic intervention studies in AN (Mack et al. 2016; Mack et al. 2018). The inclusion of fermented foods may also be considered for future protocols. These insights may provide new leads to modulate the intestinal microbiota in order to improve treatment outcomes.

## Conclusions and future directions

Profound differences in the gut microbial composition between people with AN and normal and overweight individuals have been reported; however, further research is required to clarify whether the observed differences are a cause or consequence of AN. Thus far, only two studies have investigated the changes in gut microbial composition during nutritional rehabilitation. Nutritional treatment and successful weight gain in AN did not result in a reconciliation of their gut microbiota composition to that of non-AN comparison control groups. These limited findings suggest that AN pathophysiology—at least as it relates to the gut microbiome—may persist beyond weight restoration. The primary focus of the nutritional rehabilitation in AN is to maintain weight restoration and to improve nutritional status. However, an understanding of the effects of current nutritional rehabilitation procedures on the gut microbiome suggests the need to consider additional factors for optimal AN treatment. For example, the known effects of a diet high in saturated fat or high in prebiotic fibre on gut microbial composition and consequent physiological function may better serve to inform the composition of nutritional rehabilitation diets in AN. Importantly, gut microbiome research in this population may also improve insights into disordered eating behaviour, dysregulated appetite, and comorbid depression and anxiety. Detailed information on the specific composition of nutritional rehabilitation protocols are crucial for future research investigating post-rehabilitation microbiota. This is particularly the case given that different amounts of macronutrient intake—as well as their relative proportion in the overall diet—may exert differential effects on microbial composition. As such, future studies should include information regarding patients’ baseline pre-rehabilitation diet (e.g., calorie, fat, and fibre intakes), describe nutritional rehabilitation protocols in detail, and sample the gut microbiota repeatedly during the period of nutritional rehabilitation in AN. Studies with larger sample sizes are also warranted.
